# Dynamic Spread of Antibiotic Resistance Determinants by Conjugation to a Human-Derived Gut Microbiota in a Transplanted Mouse Model

**DOI:** 10.3390/antibiotics14020152

**Published:** 2025-02-04

**Authors:** Azam A. Sher, Charles E. Whitehead-Tillery, Ashley M. Peer, Julia A. Bell, Daniel B. Vocelle, Joshua T. Dippel, Lixin Zhang, Linda S. Mansfield

**Affiliations:** 1 Comparative Enteric Diseases Laboratory, Departments of Large Animal Clinical Sciences and Microbiology, Genetics and Immunology, East Lansing, MI 48824, USA; sherazam@msu.edu (A.A.S.); white174@msu.edu (C.E.W.-T.); apeer@unmc.edu (A.M.P.); bellj@msu.edu (J.A.B.);; 2 Comparative Medicine and Integrative Biology Graduate Program, College of Veterinary Medicine, Michigan State University, East Lansing, MI 48824, USA; 3 BEACON Center for the Study of Evolution in Action, Michigan State University, East Lansing, MI 48824, USA; 4 Department of Pharmacology and Toxicology, Michigan State University, East Lansing, MI 48824, USA; vocelled@msu.edu; 5 Departments of Epidemiology and Biostatistics and Microbiology, Genetics and Immunology, Michigan State University, East Lansing, MI 48824, USA

**Keywords:** gut microbes, antibiotic resistance, RP4 conjugative plasmid, enteric pathogens, commensal bacteria, mouse

## Abstract

Background. Antibiotic-resistant (AR) bacteria pose an increasing threat to public health, but the dynamics of antibiotic resistance gene (ARG) spread in complex microbial communities are poorly understood. Conjugation is a predominant direct cell-to-cell mechanism for the horizontal gene transfer (HGT) of ARGs. We hypothesized that commensal *Escherichia coli* donor strains would mediate the conjugative transfer of ARGs to phylogenetically distinct bacteria without antibiotic selection pressure in gastrointestinal tracts of mice carrying a human-derived microbiota with undetectable levels of *E. coli*. Our objective was to identify a mouse model to study the factors regulating AR transfer by conjugation in the gut. Methods. Two donor *E. coli* strains were engineered to carry chromosomally encoded red fluorescent protein, and an ARG- and green fluorescent protein (GFP)-encoding broad host range RP4 conjugative plasmid. Mice were orally gavaged with two donor strains (1) *E. coli* MG1655 or (2) human-derived mouse-adapted *E. coli* LM715-1 and their colonization assessed by culture over time. Fluorescence-activated cell sorting (FACS) and 16S rDNA sequencing were performed to trace plasmid spread to the microbiota. Results. *E. coli* LM715-1 colonized mice for ten days, while *E. coli* MG1655 was not recovered after 72 h. Bacterial cells from fecal samples on days 1 and 3 post inoculation were sorted by FACS. Samples from mice given donor *E. coli* LM715-1 showed an increase in cells expressing green but not red fluorescence compared to pre-inoculation samples. 16S rRNA gene sequencing analysis of FACS GFP positive cells showed that bacterial families Lachnospiraceae, Clostridiaceae, Pseudomonadaceae, Rhodanobacteraceae, Erysipelotrichaceae, Oscillospiraceae, and Butyricicoccaceae were the primary recipients of the RP4 plasmid. Conclusions. Results show this ARG-bearing conjugative RP4 plasmid spread to diverse human gut bacterial taxa within a live animal where they persisted. These fluorescent marker strategies and human-derived microbiota transplanted mice provided a tractable model for investigating the dynamic spread of ARGs within gut microbiota and could be applied rigorously to varied microbiotas to understand conditions facilitating their spread.

## 1. Introduction

The emergence and global spread among bacterial pathogens of antimicrobial resistance (AMR) to life-saving drugs have become a major challenge in clinical settings. Approximately 4.95 million AMR-associated deaths occurred globally in 2019, including 1.27 million deaths directly attributed to drug-resistant bacteria according to clinical records [1]. Based on a 2019 report by the Centers for Disease Control and Prevention (CDC), more than 2.8 million infections and 35,000 deaths caused by antibiotic-resistant (AR) bacteria occur in the United States every year [2]. If antibiotic resistance concerns are not imminently addressed, AR bacteria could become a leading cause of death. In fact, estimates of up to 10 million additional AR infections globally by 2050 are expected to significantly increase the economic burden of such infections by up to USD 10 trillion [3]. In clinical settings, AR bacteria cause infections with lengthier hospitalizations and higher mortality than non-resistant bacteria [2,4] and thus have become a critical public health concern. Most AR bacteria reported in the USA and worldwide are either enteric pathogens or organisms capable of living in the human gut [1,2]. Ten of the twelve most common bacteria causing fatal AR infections, *Escherichia coli*, *Staphylococcus aureus*, *Klebsiella pneumoniae*, *Streptococcus pneumoniae*, *Pseudomonas aeruginosa*, *Enterococcus faecium*, *Enterobacter* spp., *Streptococcus agalactiae* (Group B *Streptococcus*), *Salmonella* Typhi, and *Enterococcus faecalis*, can persist in the gastrointestinal (GI) tract.

The spread of transferable ARGs among enteric pathogens, opportunistic pathogens, and commensal gut bacteria is a serious concern [5]. Every year, more multi-drug-resistant bacteria emerge [2]. Bacteria often develop antibiotic resistance by undergoing spontaneous genomic mutations followed by increases in population size because of selection pressure due to antibiotic use [6]. Detection and control efforts become more challenging when these ARGs are located on mobile elements capable of transferring them to other bacteria. Multiple studies have shown that horizontal gene transfer (HGT), which allows bacterial pathogens to acquire resistance from other bacteria, has contributed significantly to the spread of antibiotic resistance [7,8,9]. There are three main mechanisms of HGT reported in bacteria: transformation [10], transduction [11,12], and conjugation [13,14]. The human gut especially the distal bowel is densely populated by diverse bacteria and provides ample opportunity for HGT. Moreover, the gut microbiota is a known reservoir of ARGs [15,16], thus, its members are potential donors and/or recipients of ARG transfer. Additionally, given the frequent presence of opportunistic bacteria and transient pathogens in this compartment, conjugation in the human gut microbiota would arguably have the most direct impact on the spread of ARGs to previously susceptible pathogens in the body. Conjugation is considered to have the biggest impact on HGT in the gut because it requires direct cell-to-cell contact. In the gut, this is facilitated by high bacterial density and the close proximity of diverse species, allowing for the efficient transfer of plasmids between bacteria of different species. This is in contrast to HGT mechanisms like transformation, which relies on free DNA in the environment or transduction, which requires a virus intermediary. Numerous laboratory studies and genomic surveillance data support the occurrence of conjugation in different microbiotas, but this phenomenon was primarily studied in vitro or in environmental niches such as soil, animal manures, and wastewater treatment plants [17,18].

Despite the fact that the gut microbiota in humans and animals is a known reservoir of ARGs [15], little is known about the baseline transfer frequencies of ARGs, the potential reservoir of bacterial hosts of transferable ARGs, and the drivers of conjugation among these bacteria. The inability to track conjugation events in the gut, difficulty in identifying and characterizing non-culturable plasmid-recipient bacteria, and the impracticality of controlled experiments in humans has hampered research efforts to answer these questions. Recently, holistic models were employed utilizing mice carrying low-diversity defined Schaedler flora [19] subjected to pre-treatment with antibiotic [20,21] or mice carrying conventional murine microbiotas with or without selection pressure from antibiotic administration [22,23,24]. However, studies without antibiotic-induced stress are difficult to conduct. Ott and colleagues (2022) pointed out that the resistance of mice with conventional mouse microbiota to colonization with *Enterobacteriaceae* spp. conjugative donors of interest such as human gastrointestinal pathogens may be due to the requirement for anaerobiasis and the expression of stress response regulators such as ArcA, CpxR, and RcsB [25]. Likewise, Tao et al. (2022) speculated that the high density of obligate commensal anaerobic Firmicutes inhabiting the distal gut functionally blocks HGT among low density Enterobacteriaceae under normal conditions [26]. Regardless, metagenomic studies remain the main source of our current knowledge of HGT in the human gut microbiota (reviewed by Chen et al., 2020; Brito, 2021) [27,28].

**Figure 1:**
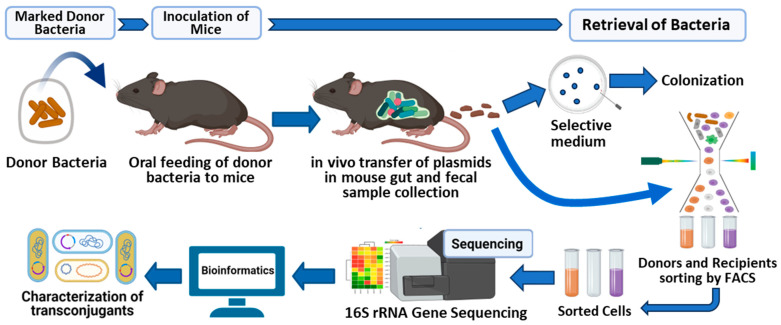
This graphical abstract shows the experimental design and methods used in this study. The mice transplanted with human adult fecal microbiota were fed fluorescently labeled donor *E. coli* bacteria carrying the GFP-encoded RP4 plasmid. The colonization of donor bacteria was assessed by culturing bacteria directly from mouse fecal samples. FACS was performed on fecal samples to detect and sort transconjugant bacteria expressing only GFP encoded on the RP4 plasmid. 16S rRNA amplicon sequencing was carried out on FACS bacteria to characterize the potential recipients of the broad host range RP4 plasmid in the gut microbiota. Created with BioRender.com.

We hypothesized that a broad host range RP4 plasmid carried by a commensal donor bacterium *E. coli* LM715-1 would be transferred to resident bacteria of the human gut microbiota. To address this hypothesis, we used the offspring of germ free C57BL/6 mice transplanted with an adult human-derived gut microbiota (Adult C) [29]. The offspring of the transplanted mice were shown to carry a stable microbial community where *E. coli* were undetectable over 30 generations. We inoculated Adult C mice carrying this human-derived infant gut microbiota [29] with previously constructed fluorescently labeled *E. coli* strains carrying the RP4 plasmid to test their ability to colonize and act as conjugative donors. These two strains included (1) a laboratory strain of *E. coli* MG1655 and (2) commensal *E. coli* LM715-1 (UPEC) isolated from mice carrying an infant-derived gut microbiota [30]. The RP4 plasmid and the chromosomes of these *E. coli* strains were both marked with distinct fluorescent markers to allow for tracking of the bacterial strains and the RP4 plasmid within the host microbiota. The RP4 conjugative plasmid is used frequently to study conjugation in mouse models because of its broad host range, allowing it to transfer genetic material to a diverse range of bacteria present in the mouse gut microbiome. This makes it ideal for investigating horizontal gene transfer dynamics within a complex microbial community. Moreover, the molecular basis of the conjugation machinery is well characterized, providing a reliable system for studying the mechanisms of plasmid transfer in vivo. Then, we employed high-throughput cell sorting, 16S rRNA amplicon sequencing, and culture to track the fluorescently labeled donor and transconjugant bacteria from fecal samples of Adult C C57BL/6 mice exposed to donor bacterial strains. The experimental design appears in Figure 1. We found that the labeled commensal donor *E. coli* LM715-1 strain persistently colonized Adult C mice throughout the ten-day experiment without antibiotic treatment. Conjugative transfer to the human-derived bacterial community occurred. The broad host range RP4 plasmid carried by the donor *E. coli* strain invaded diverse bacterial families of human gut microbiota members. Overall, these findings suggest that using a human gut-transplanted mouse model combined with high-throughput sorting and sequencing techniques are an innovative way to investigate the spread of transferable ARGs through HGT in the gut microbiota. Thus, in these experiments, we provide a robust and tractable mouse model with a stable and vertically transmissible human-derived fecal microbial community to study the spread of ARGs by conjugation and other mechanisms to a human gut microbiota.

## 2. Results

### 2.1. Validation of Murine Model Carrying Human-Derived Gut Microbiota for These Experiments

For this work, we used offspring of germ free C57BL/6 mice given human-derived fecal transplants from healthy young adults (Adult C FMT mice) that were documented to carry a *Bacteroides*–Lachnospiraceae dominant gut microbiota with undetectable levels of *E. coli* based on 16S sequencing [31]. This model was shown to undergo stable transfer of this microbiota to offspring over 35 generations when housed in a barrier facility and handled only by trained workers wearing surgical personal protective equipment. In the current study, the offspring of these mice were confirmed to have no coliform bacteria or bacteria carrying specific antibiotic resistance phenotypes critical for these experiments by culturing fecal samples on antibiotic selective MacConkey and Mueller Hinton agar plates. Fecal pellets of eight mice carrying the adult human-derived microbiota were suspended in sterile phosphate-buffered saline (PBS)(1:10 *w*/*v*). Suspended fecal pellets were serially diluted in sterile water and plated on MacConkey and Mueller Hinton agar with and without antibiotics. No colonies were obtained from fecal slurries on Muller Hinton agar containing 50 µg/mL ampicillin (AmpR), 20 µg/mL chloramphenicol (CamR), 4 µg/mL ceftriaxone (5 mice), or 50 µg/mL ampicillin (3 mice). The lower limit of detection was 100 colony-forming units (CFUs)/50 mg fecal pellet. For all eight mice, the colonies were obtained on Mueller Hinton agar without antibiotics but not MacConkey agar without antibiotics, confirming that the bacteria in these samples do not have the characteristics of *E. coli*, which are Gram-negative, grow on MacConkey’s, and are pink in color because they ferment lactose. Thus, these mice with human-derived Adult C fecal microbiota provide a model GI tract with a complex microbiome containing no detectable coliform competitors for our experimental conjugative plasmid donor *E. coli* strains and no resident antibiotic resistance determinants that could interfere with our selective plating design.

### 2.2. Fluorescently Labeled Commensal Donor E. coli Strain Colonized the Gut Longer than a Similarly Labeled Laboratory Strain of E. coli

In previous work we developed a set of marked donor strains to perform traceable in vivo conjugation experiments to document the transfer to members of the gut microbiota and confirmed conjugative transfer in vitro [32]. Briefly, we isolated the human-derived and mouse gut-adapted commensal *E. coli* LM715-1 strain from offspring of C57BL/6 mice transplanted with a mixed fecal slurry from five 3-month-old human infants (Infant B). We also verified that *E. coli* LM715-1 was susceptible to the antibiotics ampicillin (50 µg/mL), kanamycin (50 µg/mL), chloramphenicol (20 µg/mL), tetracycline (15 µg/mL), and ceftriaxone (4 µg/mL) [30]. Thereafter, *E. coli* LM715-1 was chromosomally labeled with a gene cassette encoding chloramphenicol (*CamR)* and kanamycin (*KanR*) antibiotic-resistant markers as well as the mScarlet fluorescent protein (Sher et al. (2023) [32].

**Figure 2:**
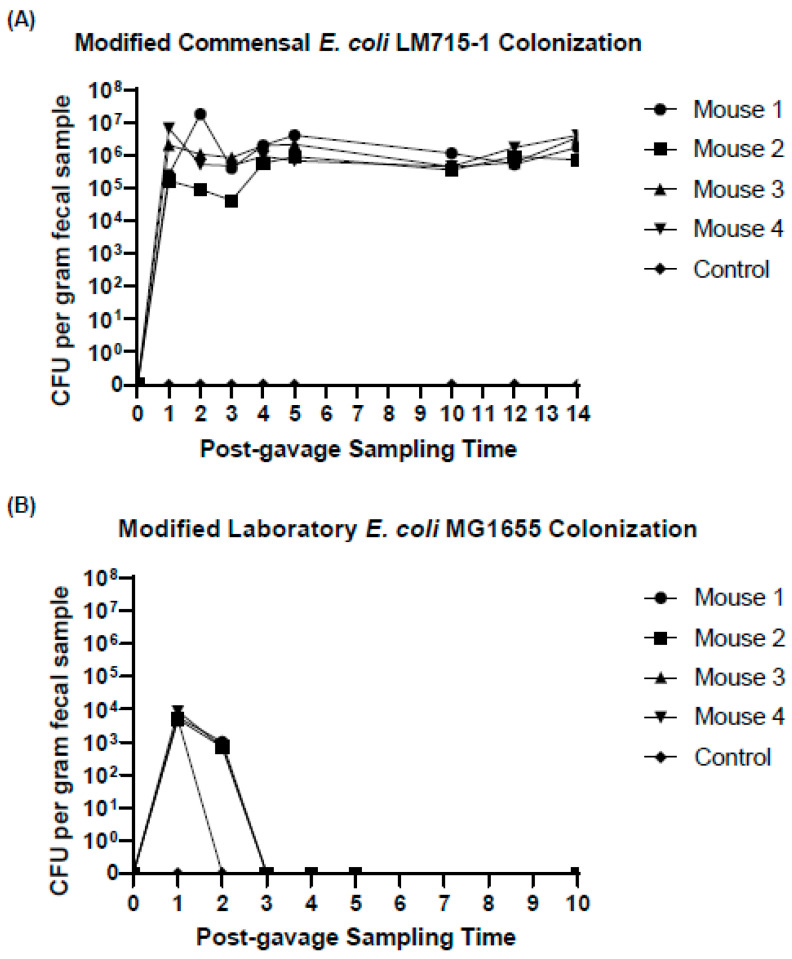
The colonization of the fluorescently labeled commensal and laboratory strains in the mouse gut. (**A**) shows the total colony-forming units (CFUs) of the human-derived *E. coli* LM715-1 bacterium identified throughout the 14-day experiment as determined by serial dilution plating on MacConkey agar containing chloramphenicol. (**B**) shows the colonization of the labeled laboratory strain of *E. coli* MG1655 in the mouse gut as determined by serial dilution plating on MacConkey agar containing ampicillin. Each symbol represents an individual mouse (*n* = 4). The lower limit of detection was 100 CFUs per gram fecal material.

To assess the persistence of chromosomal markers in commensal human *E.* coli LM715-1, we orally inoculated four mice carrying Adult C human-derived gut microbiota with this modified *E. coli* LM715-1 strain without the labeled plasmid and without applying antibiotic selective pressure. Two mice were sham-inoculated with phosphate-buffered saline (PBS) to serve as negative controls. Fecal pellets were then collected daily from all mice for 14 days and screened for chloramphenicol and kanamycin antibiotic-resistant phenotypes using antibiotic selective plating on MacConkey agar. The presence of mScarlet fluorescent protein was confirmed using fluorescence microscopy and colony PCR as described in Sher et al. (2023) [32]. We found that the marked commensal *E. coli* LM715 strain successfully colonized the gut of all mice without applying antibiotic selection pressure (Figure 2A). This screening also confirmed that all three markers (*mScarlet*, *CamR*, *KanR*) inserted on the chromosome were maintained without applying antibiotic selective pressure. We also inserted the gene cassette into the laboratory strain, *E. coli* MG1655, and tested its colonization of Adult C microbiota mice in an identical experiment. We found that the labeled laboratory strain of *E. coli* MG1655 did not colonize for longer than 72 h (Figure 2B). However, the *E. coli* MG1655 did maintain fluorescent and antibiotic-resistant markers during transit in the gut. These findings showed that using either strain as a donor was possible but that the commensal mouse gut-adapted *E. coli* LM715-1 would be more useful than *E. coli* MG1655 for studying horizontal gene transfer for an extended period, especially the plasmid-mediated spread of ARGs in the gut microbiota in live animals.

### 2.3. Commensal Donor E. coli LM715-1 Strain Carrying the RP4 Plasmid Persisted in the Gut Without Antibiotic Treatment

The broad host range plasmid RP4::GFP, *amp*R, *tet*R, *kan*R was then introduced into *E. coli* LM715-1 *mScarlet*, *Cam*R, *Kan*R by conjugation using donor strain *E. coli* MG1655, a kind gift from Professor Bart F. Smets (University of Aarhus, DK) [18,32]. Details of this procedure were previously published [32]. Recipients were isolated by jointly selecting for antibiotic-resistant markers harbored by the RP4 plasmid and those harbored by the chromosomally marked *E. coli* LM715-1; we could also distinguish the *E. coli* LM715 recipients that acquired the RP4 plasmid due to the expression of the plasmid-borne GFP and the chromosomally borne mScarlet protein.

**Figure 3:**
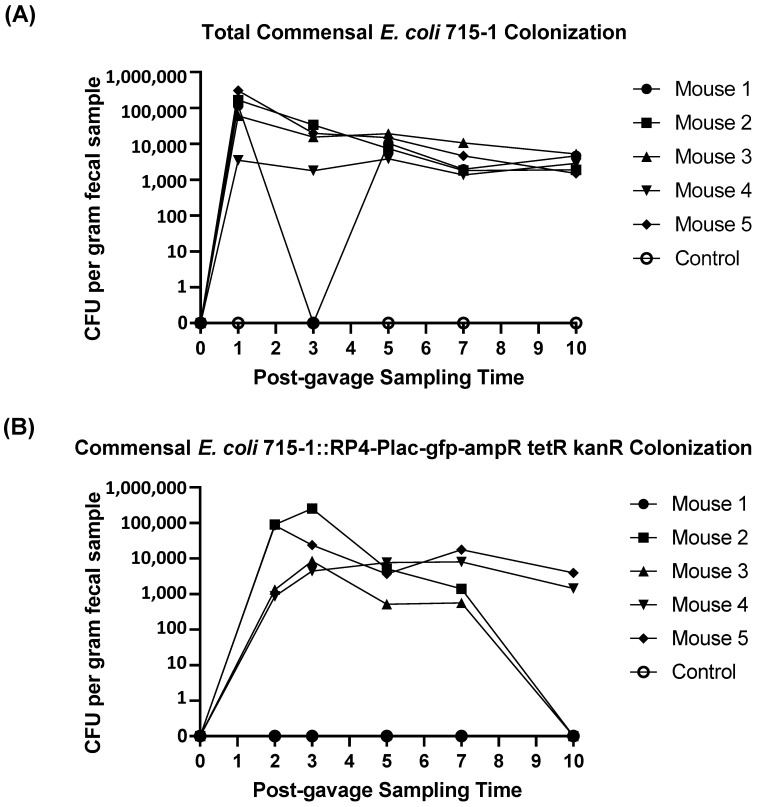
A modified commensal donor *E. coli* strain colonized the mouse gut in the absence of antibiotic selection pressure. (**A**) The total number of bacteria with and without RP4 plasmid is shown for each mouse as determined by serial dilution plating on MacConkey agar containing chloramphenicol. (**B**) The number of bacterial cells carrying RP4 plasmid (donor bacteria) on different days of the ten-day experiment was determined by serial dilution plating on MacConkey agar containing chloramphenicol and ampicillin. Each symbol represents an individual mouse (*n* = 5). Mouse 1 remained negative for donor bacteria after 24 h. Two mice (*n* = 2) were in the control group given Luria broth only. The lower limit of detection was 100 CFUs per gram of fecal material.

Thereafter, we tested whether the mouse gut-adapted commensal *E. coli* LM715-1 carrying RP4 had the ability to colonize the gut of mice with human-derived Adult C fecal microbiota and whether the plasmid would be maintained without applying antibiotic selection pressure. The chromosomally labeled *E. coli* LM715-1 carrying the RP4 plasmid was given by oral gavage to five mice, and fecal samples were collected for ten days with a sham-inoculated sixth mouse to serve as a negative control. None of these mice were given antibiotics. Fecal samples from mice were screened for the total number of chromosomally labeled *E. coli* 715-1 either with or without the RP4 plasmid by suspending fecal pellets in PBS (1:10 *w*/*v*), serially diluting them in sterile water, and plating them on selective MacConkey agar medium containing chloramphenicol. We determined the numbers of chromosomally labeled *E. coli* carrying the RP4 plasmid by simultaneous plating on MacConkey agar containing chloramphenicol to identify the *E. coli* LM715 chromosome and ampicillin to identify the RP4 plasmid. All five mice were colonized with *E. coli* LM715-1 carrying chloramphenicol resistance throughout the ten-day experiment (Figure 3A). The chromosomally labeled donor strain *E. coli* LM715-1 carrying RP4 was recovered from 80% of mice (4/5) on day 7 and 40% (2/5) on day 10. Furthermore, *E. coli* LM715-1 carrying RP4 maintained resistance to chloramphenicol and ampicillin without any antibiotic selection pressure for plasmid retention during colonization of the mice (Figure 3B). These data suggest a fitness cost associated with the carriage of the RP4 plasmid, which ultimately caused the loss of the plasmid in some mice over ten days, such that donor cells that experience segregational loss of this large, low-copy number plasmid have a selective advantage in the gut environment over those that retain it.

**Figure 4:**
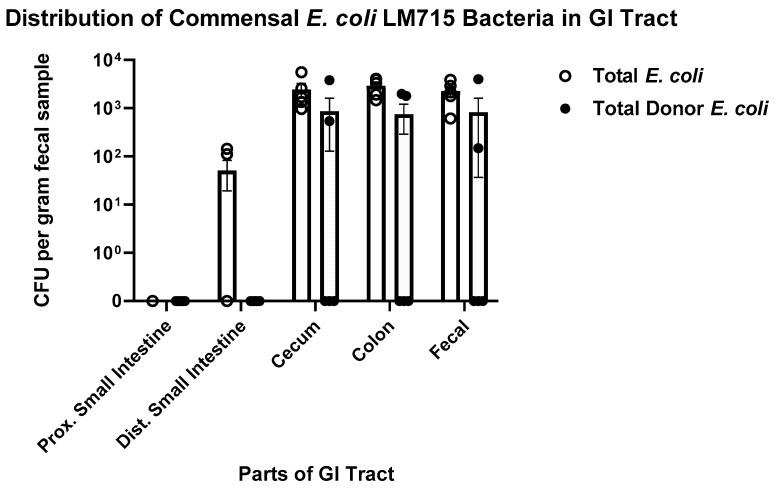
The distribution of the modified commensal donor *E. coli* strain in the mouse gut at the end of the ten-day experiment. The open circles show the total number of bacterial cells with and without RP4 plasmid as determined by serial dilution plating on MacConkey agar containing chloramphenicol, while the filled circles show the mice with donor bacteria carrying RP4 plasmid as determined by serial dilution plating on MacConkey agar containing chloramphenicol and ampicillin. Each symbol represents an individual mouse in the group (*n* = 5); 100 CFUs per gram fecal material was the lower limit of detection.

At the end of the ten-day experiment, we also determined the spatial distribution of the labeled *E. coli* LM715-1 strain in different parts of the GI tract (Figure 4). The labeled *E. coli* LM715-1 strain largely colonized distal parts of the GI tract: cecum and colon. The total population sizes of chromosomally labeled LM715-1 with and without RP4 ranged from 10^3^ to 10^4^ CFU of bacterial cells per gram of intestinal contents. The same level was detected in fecal samples collected before necropsy on day 10 (Figure 4). Donor *E. coli* cells carrying the RP4 plasmid were isolated from both cecum and colon in two out of five mice with population sizes ranging from 10^2^ to 10^3^ CFU bacterial cells per gram of intestinal content (Figure 4). These findings showed that the commensal chromosomally labeled *E. coli* strain mainly colonized the cecum and colon and that fecal samples were a good proxy for assessing colonization.

### 2.4. Determination of FACS Gating Strategy with Commensal Donor E. coli LM715-1 Before Inoculation into Mice

The presence of unique fluorescent markers on the donor *E. coli* LM715-1 chromosome (mScarlet) and the transferable RP4 plasmid (GFP) enabled us to distinguish transconjugant bacterial strains that acquired RP4, which would exhibit green but not red fluorescence due to the presence of GFP. Alternatively, donor *E. coli* LM715-1 strains carrying RP4 or that had lost RP4 would show either the presence of both the mScarlet protein and GFP or only the mScarlet protein, respectively, in flow cytometry. We determined the gating strategy using control bacterial culture samples with and without fluorescent markers by following methods described previously [18,33]. In particular, we used the two-stage sorting and gating strategy described by Klumper et al. (2015) to minimize the effect of autofluorescence [33].

**Figure 5:**
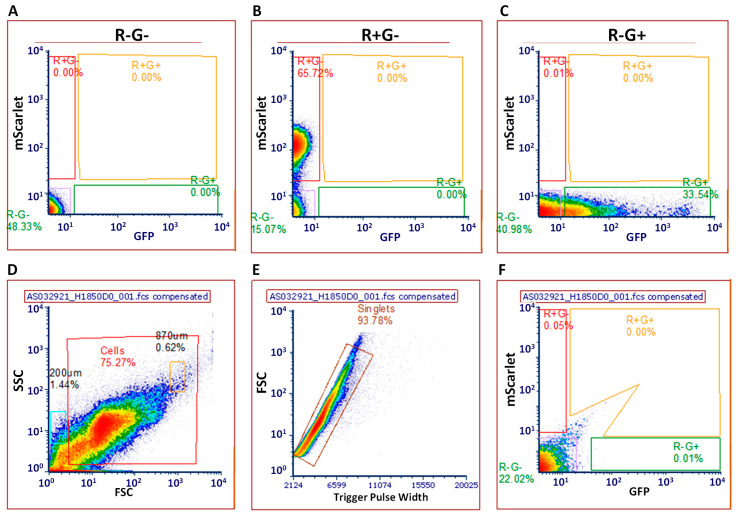
Flow cytometric sorting of bacterial cells with and without fluorescent proteins. Gates were drawn based on pure cultures of bacteria: (**A**) *E. coli* LM715-1 bacteria without either fluorescent protein (R−G−), (**B**) *E*. *coli* LM715-1 bacteria with mScarlet (R+G−), and (**C**) *E. coli* LM715-1 bacteria with GFP (R−G+). Panels (**D**–**F**) show flow cytometric sorting of bacterial cells from control mouse fecal sample to demonstrate background fluorescence. Panel (**D**) shows 75.27% of bacterial cells, panel (**E**) shows singlets, and panel (**F**) shows sort-I to select red only gate (0.05%), green only gate (0.01%), and red and green gate (0.00%). Note that background values are exceedingly low.

Panels A-C of Figure 5 illustrate the gates for identifying and sorting bacteria with no fluorescence (R−G−), red fluorescence only (R+G−), green fluorescence only (R−G+), and both red and green fluorescence (R+G+). About 48.33% of the *E. coli* LM715-1 cells did not exhibit any fluorescence (R−G− gate) when the markers were not present (Figure 5A). When *E. coli* LM715-1 contained only the mScarlet protein, 65.72% of the cells were present within the R+G− gate (Figure 5B). In Figure 5C, 33.54% of the *E. coli* LM715-1 cells that contain the RP4::GFP but not the mScarlet protein exhibit fluorescence in the R−G+ gate.

After determining the gating strategy using pure bacterial cultures, fecal samples from the uninoculated mice were examined using FACS to determine the amount of autofluorescence due to the microbiota. The fecal samples were processed to remove organic debris by a method modified from Ronda et al. (2019) that comprised repeated washes with low-speed centrifugation followed by filtration through a 40 µM filter [34]. Trial sorting of fecal samples revealed that 27.76% of the cell population was recovered (Figure 5D). Of the 27.76% cells recovered, 98.02% are individual cells or singlets (Figure 5E). However, we detected autofluorescence in 0.08% of the cells in the R+G− gate, 0.04% in the R+G+ gate, and 1.01% in the R−G+ gate when the commensal donor *E. coli* LM715 was uninoculated (Figure 5F). Overall, these results suggest that our fluorescent markers are detectable by flow cytometry; however, the autofluorescence from the fecal sample may impede our ability to distinguish true transconjugant cells from autofluorescent cells.

### 2.5. Sorting of Transconjugant Bacteria That Received Labeled RP4 Plasmid from Commensal Donor E. coli LM715-1

**Figure 6:**
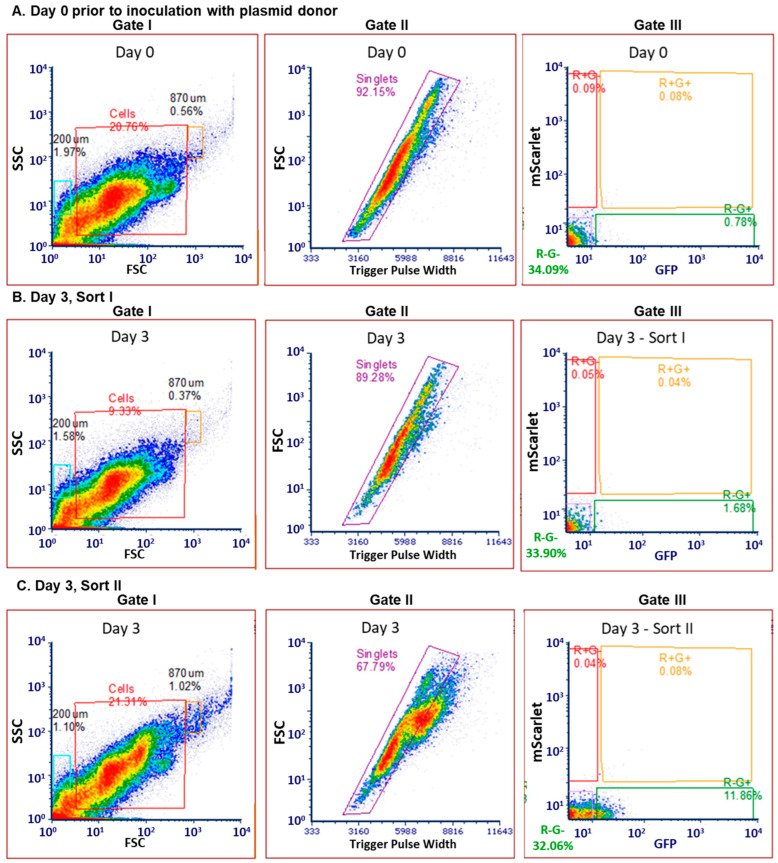
FACS of transconjugant bacteria from fecal samples of mice inoculated with modified commensal donor *E. coli* strain carrying the RP4 plasmid. Sorting was performed using three successive gates shown in three columns (I, II, III). Gate I sorts for bacterial size based on forward scatter (FSC) and side scatter (SSC); gate II sorts for singlets; and gate III sorts cells expressing green only, red only, green and red, and no fluorescence. (**A**) First row shows sorting of fecal samples collected on day 0 before inoculation of mice with the donor strain. Autofluorescence was detected in a drawn gate for GFP while sorting fecal samples before inoculation. Two consecutive sorts were performed to minimize autofluorescence. (**B**) Second row shows first sorting (sort-I) of cells from fecal samples collected on day 3 (72 h after inoculation). (**C**) Third row shows second sorting of previously sorted GFP positive cells in sort-I to select only those green cells that display only green and no red fluorescence. These two consecutive sorts (I, II) reduced chances of false-positive cells and effects of autofluorescence.

To reduce interference from autofluorescent cells in sorting the experimental samples, we used the two-stage sorting strategy described by Klumper et al. (2015) [18] so as to detect true transconjugant bacteria expressing only GFP in post inoculation samples. Before inoculating the mice with the *E. coli* LM715-1 *mScarlet*, *Cam*R, *Kan*R carrying the RP4:: RP4::GFP, *amp*R, *tet*R, *kan*R plasmid, we sorted a fecal sample at day 0 using FACS. We recovered 20.76% of the cells from the fecal sample in gate 1 with 92.15% being individual cells in gate II (Figure 6A). At day 0, an autofluorescent signal was detected at about 0.78% of the cells in gate III (Figure 6A). After inoculating the mice with the *E. coli* LM715-1 carrying the RP4 plasmid, we selected two time points, 24 and 72 h post inoculation, for the detection of transconjugant bacteria in fecal samples from experimentally inoculated mice. We carried out fluorescence-activated two-stage cell sorting (FACS) on fecal samples collected on day 1 and day 3 post inoculation of Adult C microbiota mice with donor strain *E. coli* LM715-1 carrying plasmid RP4::GFP, *amp*R, *tet*R, *kan*R. About 200,000 transconjugant bacteria expressing RP4 plasmid-encoded GFP were FACS and collected from each of the five mice on day 1.

For sort I on day 3 post inoculation, we recovered 9.33% of cells in gate I with 89.28% being individual cells in gate II. Furthermore, in gate III, 1.68% of the cells fell in the green fluorescence only gate (Figure 6B). Those cells expressing only GFP were collected and sorted a second time to obtain pure green fluorescent only cells and exclude other bacteria; green fluorescent only bacteria now comprised 11.86% of the sorted sample in gate III (Figure 6C). However, we only recovered 21.31% of cells in gate I with 67.79% being individual cells in gate II (Figure 6C). These two consecutive sorts reduced the chances of false-green, fluorescent-only positive cells and the effect of autofluorescence. On day 3, three fecal samples yielded around 200,000 FACS cells, and two samples yielded 101,000 and 69,000 cells. In addition, we collected about 7.69 × 10^5^ total cells from the five mice on day 3 after the two FACS. The fraction of donor cells (both red and green fluorescence) remained <1% throughout. These findings support the conclusion that the RP4 plasmid was transferred from donor *E. coli* LM715-1 to gut resident bacteria, which then expressed plasmid-encoded GFP.

### 2.6. FACS Putative Transconjugants Comprised Diverse Population of Human Gut Microbiota Members

**Figure 7:**
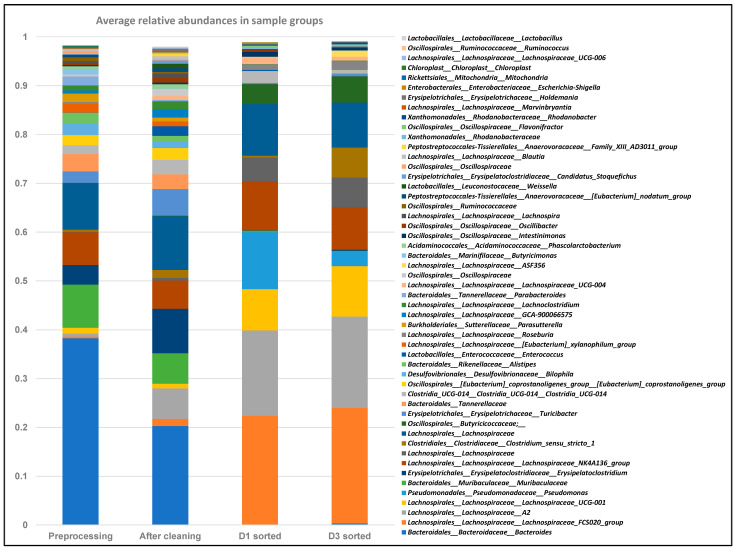
Relative abundance of fecal bacterial taxa in FACS bacteria based on 16S rRNA gene sequencing. Y-axis represents average relative abundance of 49 bacterial operational taxonomic units (OTUs) identified in fecal samples of five mice at relative abundance of at least 0.5% in at least one sample. Different time points where 16S sequencing analyses were carried out are shown on X-axis. The “pre-inoculation” stacked bar shows reference average gut microbial community before inoculating mice with donor bacterium *E. coli* LM715-1 carrying RP4 plasmid. The “D1 after cleaning” stacked bar shows average day 1 microbial community before FACS but after washing fecal samples. The “D1 sorted” and “D3 sorted” stacked bars show average relative abundances of putative transconjugant bacteria identified on day 1 and day 3 post inoculation of mice with donor *E. coli* LM715-1 after two-stage sorting, as described in the Section 4. Mitochondrial and chloroplast sequences were eliminated from the dataset.

We hypothesized that after introducing the labeled plasmid-bearing commensal *E. coli* LM715-1 donor bacterium orally to mice, the conjugative RP4 plasmid encoding ARGs would spread among resident bacteria of the gut microbiota. After FACS of transconjugant bacteria, we performed 16S rRNA amplicon sequencing to characterize the bacteria that acquired the plasmid in the mouse gut. DNAs isolated from pre-inoculation fecal samples before and after removal of debris (controls) and FACS-sorted green fluorescent cells from each of five mice from days 1 and 3 were sequenced individually, resulting in 23,123–29,890 sequences per sample. These 16S sequences were analyzed using the Qiime2 pipeline to identify operational taxonomic units (OTUs) in the pool of potential transconjugant bacteria. The 16S sequencing analysis showed that the RP4 plasmid effectively invaded diverse members of this adult human-derived gut microbiota. Moreover, 115 unique OTUs were identified in the FACS-sorted putative transconjugant pool on day 1 and 137 OTUs on day 3. Of these OTUs, the 49 bacterial taxa present at a relative abundance of at least 0.5% in at least one sample are shown in Figure 7. Large differences in the relative abundances of the genus *Bacteroides* and several members of the family Lachnospiraceae are evident in the sorted samples. OTUs representing recipients of the RP4 plasmid found on day 1 were also largely present on day 3, showing that these recipient bacteria harbored the plasmid after 72 h (Figure 7). Members of bacterial families Lachnospiraceae, Clostridiaceae, Pseudomonadaceae, Rhodanobacteraceae, Erysipelotrichaceae, Oscillospiraceae, and Butyricicoccaceae were the leading recipients of the RP4 plasmid (Figure 7). We found that the RP4 plasmid invaded 16 different OTUs classified as belonging to the bacterial family Lachnospiraceae, notably those designated A2, ASF356, FCS020, and UCG-004, in addition to an OTU simply designated “Lachnospiraceae”. Thus, this family was the most common recipient taxon for the RP4 plasmid-mediated transfer of ARGs.

**Figure 8:**
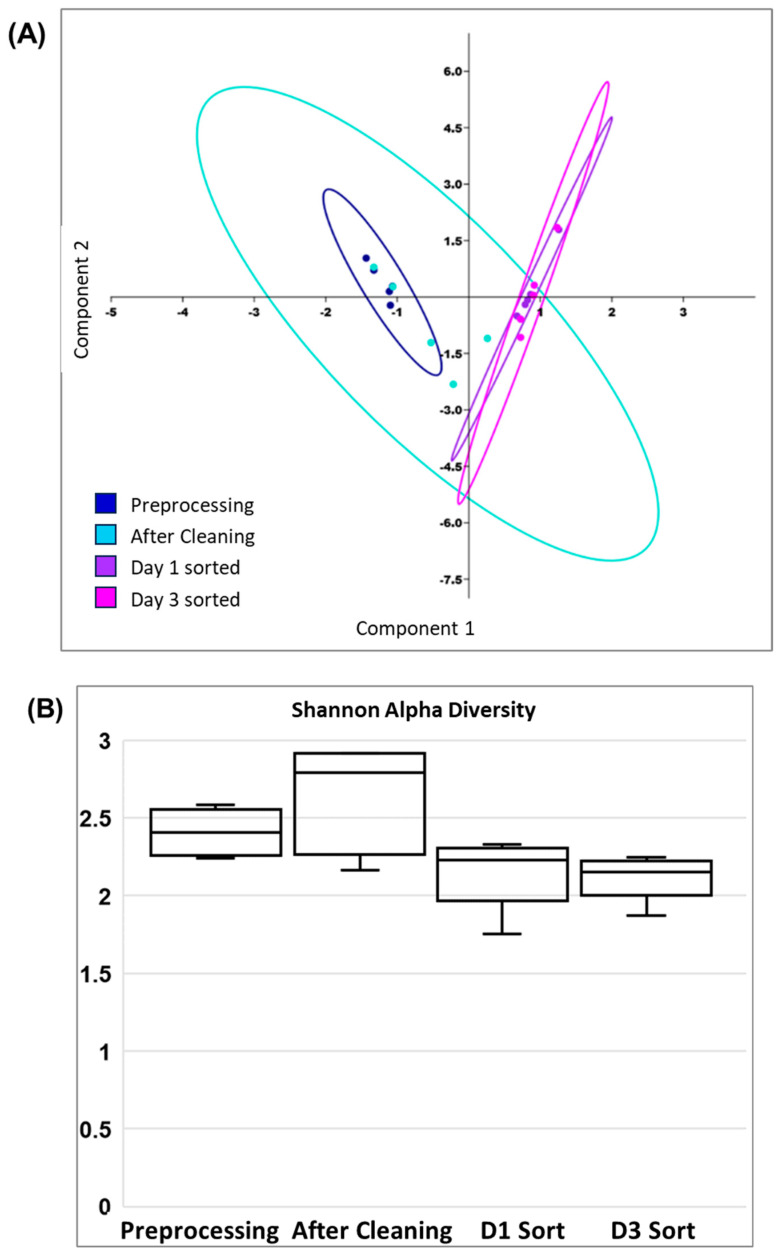
Principal component analysis (PCA) and Shannon alpha diversity of samples at different stages of processing and colonization: preprocessing, post washes, day 1 after two-stage sorting, day 3 after two-stage sorting. (**A**) describes PCA1 and PCA2 of taxon relative abundances from 16S rRNA gene sequencing data. PCA axis 1 accounts for 69.3% and PCA axis 2 captures 9.1% of variation among different samples. (**B**) Shannon diversity of gut microbial communities in fecal samples collected at different stages.

Principal component analyses also showed that the populations of sorted cells from day 1 and day 3 were similar and grouped together, while pre-inoculation samples and day 1 post-cleaning samples were grouped together in PCA (Figure 8A); the genus *Bacteroides* and two members of the family Lachnospiraceae, those designated A2 and FCS020, contributed the major loadings on PCA axis 1. The Shannon diversity index showed an apparent increase in diversity after the removal of debris and contaminants. The day 1 and day 3 sorts show that the diversity index dropped, indicating that only certain microbial populations were detected as recipients of the RP4 plasmid (Figure 8B). Overall, these findings indicate that a broad host range RP4 plasmid encoding beta-lactamase and other ARGs invaded a diverse population of resident bacteria in this Adult C human-derived gut microbiota.

## 3. Discussion

In this study, we used a mouse model [31] that carried a human-derived gut microbiota combined with a traceable mouse-adapted commensal donor *E. coli* strain to study the conjugative plasmid-mediated spread of ARGs among resident bacteria of the gut. This transplanted inbred C57BL/6 mouse model was shown to stably carry this Adult C gut microbiota over 20–35 generations [35], allowing for experimental reproducibility and offering the opportunity to address questions related to HGT.

In past studies, computational genomic methods were used to identify and confirm ARG transmission among pathogens and resident bacteria of the gut microbiota [20,27,36,37]. Some studies have also reported the plasmid-mediated transfer of ARGs in animal models including conventional mice [19,34,38,39], chickens [40], zebrafish [41], and pigs [42]. To our knowledge, this is the first study using mice carrying a stable human-derived gut microbiota to examine conjugative transfer in a complex community; this model provides a practical means of investigating the spread of ARGs via conjugation and other mechanisms of HGT.

Studying the spread of antibiotic resistance in the gut microbiota of healthy individuals is as important as studying it in patients with gastroenteritis or other diseases. One study has shown that ARGs in healthy people remained persistent and resilient to short-term changes in gut microbiota [43]. Therefore, we used a commensal human gut-derived donor *E. coli* LM715-1 strain to investigate plasmid spread in a mouse model colonized with a human-derived gut microbiota without applying antibiotic selection pressure or inducing any unhealthy or stressful conditions in the host. Our work showed that commensal donor bacteria *E. coli* LM715-1 persisted in the gut throughout the ten-day experiments without the administration of antibiotics, while the laboratory donor *E. coli* MG1655 strain did not colonize for more than 3 days. Nevertheless, there was an apparent fitness cost to the maintenance of the labeled RP4 plasmid in the donor strain, which resulted in a decrease in the potential donor population. The possible influence of plasmid maintenance on donor fitness is a phenomenon that is likely to affect the degree of plasmid spread in natural settings.

Finally, we observed that a broad host range RP4 plasmid spread from the labeled commensal donor *E. coli* LM715-1 strain to diverse members of this human-derived Adult C gut microbiota. 16S sequencing analysis of FACS-sorted cells showed Lachnospiraceae, Clostridiaceae, Pseudomonadaceae, Rhodanobacteraceae, Erysipelotrichaceae, Oscillospiraceae, and Butyricicoccaceae are the primary potential recipient bacterial families of RP4 plasmid. Interestingly, Ronda et al. (2019) studied the ability of genetic inserts in different lab-based conjugative plasmids, including the RP4 backbone plasmid, to spread to members of a conventional mouse gut microbiota [34]. They identified the transfer of plasmid-mediated genetic inserts in multiple genera of Lachnospiraceae, Clostridiaceae, and Pseudomonadaceae in conventional mouse gut microbiota [34]. Other previous studies have shown the transfer of RP4 plasmid from laboratory strains of *E. coli* to diverse populations of bacteria in soil [18,44], sewage, activated sludge [45,46,47], and the conventional mouse gut [34]. Here, we have shown for the first time that the RP4 plasmid potentially invaded diverse resident bacteria derived from the human gut from a commensal human donor bacteria, *E. coli* LM715-1. Longitudinal analyses also showed that most OTUs identified in the potential transconjugant pools appeared similar on days 1 and 3, suggesting that the RP4 plasmid could be maintained in members of the gut microbiota.

Definitive genetic proof of plasmid transfer awaits culturing and genetic sequencing of potential transconjugants. In this study, we were unable to isolate aerobic and microaerophilic transconjugants by directly culturing from fecal samples, possibly due to the low frequency of the plasmid transfer. Furthermore, we were unable to culture any aerobic or microaerophilic transconjugants from the green only fluorescent FACS cell fraction. The repetitive exposure of the bacteria to the high-energy laser involved in the two consecutive FACS likely killed the cells. This problem could be resolved by creating a more strongly fluorescent-tagged plasmid and collecting a distinct bright transconjugant pool after a single sort through flow cytometry, in which more cells could survive. There are many brighter fluorescent proteins available, e.g., sGFP, mScarlet, and mCardinal, that could be used in the future to create labeled donor strains and plasmids for this purpose [48].

Overall, our study findings demonstrate the use of a tractable mouse model carrying transplanted human-derived gut microbiota to study the plasmid-mediated transfer of ARGs longitudinally. The use of models such as this one could alleviate the impracticality of human studies of conjugation and other horizontal gene transfer mechanisms among human gut microbiotas and allow for better understanding of mechanisms that could serve as possible critical control points. In future studies, more broad host range plasmids belonging to other incompatibility groups (IncA/C, IncL/M, IncN, IncP, IncQ, IncW) could be tested in this mouse model to understand transferable ARGs and their vectors in depth and provide more general and more clinically applicable information: for example, studies on the effect of antibiotic usage on the colonization and persistence of plasmid-bearing bacteria and the further spread of ARGs to gut resident microbiota. Integrating different research methods such as flow cytometry [49] with epic PCR [50] and single-cell sequencing [51] could also advance the study of spread of ARGs in the gut microbiota.

## 4. Materials and Methods

### 4.1. Animal Ethics Statement

All mouse experiments were conducted according to guidelines provided by the American Veterinary Medical Association (AVMA) and the National Institute of Health Guide to the Care and Use of Laboratory Animals. This protocol was reviewed and approved by the Institutional Animal Care and Use Committee of Michigan State University (06/18-080-00). All mice used in these studies were C57BL/6 mice originally obtained from The Jackson Laboratory (Bar Harbor, ME, USA) barrier facility in a single purchase. In studies conducted previously, we inoculated germ free mice with human-derived fecal microbiota from adults (Adult C microbiota mice) [29] and infants (Infant B mice) [30]. The commensal donor *E. coli* LM715-1 strain was isolated from the human infant-derived fecal microbiota of Infant B mice and engineered for fluorescent labeling of the chromosome, as described previously [32]. In these manipulations, we limited the number of passages of this strain in vitro before placing it back into the experimental mice.

All experiments were conducted with age-matched male and female mice between 8 and 10 weeks of age from a breeding colony established by Adult C fecal transplantation of germ-free mice [29]. Specific pathogen free breeding colonies for these mice were established on the campus of Michigan State University in facilities free of *Helicobacter*, *Campylobacter*, *Citrobacter rodentium*, *Enterococcus faecalis*, and other known colitis-causing or respiratory pathogens based on testing by IDEXX Laboratories (Westbrook, ME, USA) and as previously described [52]. Sentinel mice were used by MSU Campus Animal Resources in the Adult C humanized microbiota mouse colony to monitor for known mouse pathogens and *Helicobacter* spp. at 6-month intervals (IDEXX BioAnalytics, Columbia, MO, USA). For all mouse studies including those described in this paper, the mice were managed under biosafety level 2 regulations in sterile cages with filtered air, and with sterile food, water, and bedding. Thus, the mice had only the human-derived microbiota, which passed down unchanged to their offspring. All operators including scientists and animal care personnel were gowned and gloved using sterile technique every time the mice were handled to prevent the transfer of the operators’ or environmental microbiota to the mice.

### 4.2. Media, Chemicals, and Reagents

MacConkey agar (Neogen; Lansing, MI, USA), Bacteriological Agar (Neogen), Luria agar (Accumedia; Hillsboro, OR, USA), Luria-Miller Broth (LB, IBI Scientific; Dubuque, IA, USA), and Mueller Hinton II agar (Becton Dickinson; Franklin Lakes, NJ, USA) were used to grow donor, recipient, and transconjugant bacteria and screen fecal pellets for the presence of antibiotic-resistant bacteria. All bacterial culturing was performed aerobically or in microaerophilic conditions using a gas mixture of 80% N_2_, 10% CO_2_, and 10% H_2_ at 37 °C either in an incubator or on a shaker at 150 rpm. We used antibiotics (all from Sigma Aldrich: St. Louis, MO, USA) at the following concentrations, ampicillin (50 μg/mL), chloramphenicol (20 μg/mL), kanamycin (50 μg/mL), and tetracycline (15 μg/mL), throughout the study. We used MacConkey agar or LB agar containing chloramphenicol (20 μg/mL) and ampicillin (50 μg/mL) to select for the donor strain. LB agar containing rifampicin (20 μg/mL) was used to select for the recipient strain, and LB agar containing rifampicin (20 μg/mL) and ampicillin (50 μg/mL) was used to select for transconjugants. Phosphate-buffered saline (PBS-1X) was used for washing and diluting bacterial cultures.

### 4.3. Inoculation of Mice with Donor Strain

Based on 16S sequencing analysis, the mice carrying Adult C human-derived microbiota have a *Bacteroides*–Lachnospiraceae dominant gut microbiota with undetectable levels of *E. coli.* We also confirmed the absence of *E. coli* for these experiments by plating fecal samples on MacConkey agar collected from five mice of two generations. Thus, this human-derived gut microbiota transplanted mouse model contained no native *E. coli* that might obscure the detection of conjugative transfer of ARGs.

The labeled commensal donor strain *E. coli* LM715-1 (mScarlet, KanR, and CamR) carrying the RP4 conjugative plasmid was inoculated into Luria-Miller broth containing ampicillin and chloramphenicol and incubated overnight in a shaker at 37 °C and 150 rpm. The next day, the overnight culture was washed twice and resuspended in phosphate-buffered saline (PBS) to remove antibiotics. The optical density (OD_600_) was adjusted to 1.0 (approximately 10^9^ cells per mL) before inoculating the mice with 100 μL bacterial culture using a pipet tip and inoculating the suspension carefully into the mouth.

All experiments were conducted with singly housed mice to prevent the transfer of transconjugants between group-housed mice, thereby overestimating plasmid persistence and transfer of ARGs. Every experiment had five mice in the treatment group (donor strain) and two or three mice in the control group (PBS). Fecal pellets were collected periodically before and after inoculation, suspended in LB with 30% glycerol, and stored at −80 °C throughout the experiment. All mice were humanely euthanized at the end of the experiments by CO_2_ asphyxiation; fecal pellets and different parts of the GI tract (duodenum, jejunum, ileum, cecum, and colon) were collected at necropsy, suspended in LB with 30% glycerol, and stored at −80 °C. These stored samples were later processed to measure colonization of the donor strain by culture and perform flow cytometry to detect the donor and any other bacteria that acquired the plasmid for DNA extraction and 16S sequencing to characterize the gut microbiota.

### 4.4. Assessing Colonization of Donor Strain in Fecal and GI Samples

The stored fecal and GI samples were thawed on ice. Then, 100 μL of each sample was serially diluted in ten-fold steps in PBS and spread on MacConkey agar plates with ampicillin and chloramphenicol to select for the donor strain carrying the RP4 plasmid and MacConkey agar plates with chloramphenicol only to select for the donor strain with or without the plasmid. The plates were incubated aerobically overnight at 37 °C. Individual colonies were counted, and the colonization of the donor strain in the fecal and GI segment samples of every mouse in the treatment and control groups were calculated.

### 4.5. Screening of Human Adult Fecal Microbiota Transplanted Mice for Presence of Bacteria with Antibiotic Resistance Phenotypes

Fecal pellets were collected individually into sterile tubes from three to four group-housed mice in different cages twice from two different generations of human fecal microbiota transplanted mice. We used two growth media: MacConkey agar to select only for coliform bacteria and Mueller Hinton agar to select for a broader bacterial community. Both growth media were supplemented singly with antibiotics (kanamycin (50 μg/mL), chloramphenicol (20 μg/mL), ceftriaxone (4 μg/mL), and ampicillin (50 μg/mL)). All plates spread with fecal bacterial suspension were incubated at 37 °C for 24 to 48 h.

### 4.6. Detection of Fluorescence in Donor and Transconjugant Bacteria

Bacterial cells from the single colonies of donor and transconjugant selective media plates were suspended in PBS and centrifuged at 10,000× *g* for 1 min at room temperature in an Eppendorf centrifuge 5415D with F-45-24-11 Rotor. The pellet was resuspended in 100 μL of PBS. We used the agar pad method to obtain images of fluorescent bacteria (donor and transconjugants) [53]. Then, a 1% agarose solution was poured on a plain surface bordered with microscope slides to obtain the same thickness agar pads. After the agar solidified, coverslip size pads were cut using a sterile scalpel and placed on another microscope slide. Then, 2–5 μL of the bacterial suspension suspended in PBS was spread over the agar pad and covered with a coverslip. A Nikon eclipse N*i* microscope with bright field, GFP (excitation peak 488 nm, emission peak at 509 nm), and RFP (excitation peak 552 nm, emission peak at 583 nm) filters were used to record and analyze the fluorescent bacteria at magnifications of 20× and 40×. For rapid screening of individual fluorescent bacterial colonies, a small number of bacteria were picked with a sterile loop directly from the individual colonies grown on the plate and mixed with 10 μL of PBS or deionized water on a microscopic slide. After covering with a microscopic coverslip and air-drying for five-ten minutes, we scanned for fluorescent mScarlet and GFP expression in the donor, recipient, and transconjugants under the microscope.

### 4.7. Validation of Antibiotic Resistance Markers and Fluorescence in Donor and Transconjugants

To confirm the presence of antibiotic resistance markers in donor and transconjugant cells, we used two strategies: (1) direct plating bacterial suspension of fecal pellets on media containing specific selective antibiotic(s) and (2) colony PCR using specific primers against antibiotic resistance genes present on the plasmid and donor’s chromosome.

We performed colony PCR to confirm the presence of the RP4 plasmid in transconjugant bacteria using primers for the GFP marker located on the plasmid (Table 1). A small amount of a bacterial colony was harvested using a sterile toothpick and mixed with a 25 μL reaction mixture in PCR tubes. Each tube reaction mixture contained 2.5 μL 10× buffer (MgCl_2_ free), 2.5 μL MgCl_2_ (50 mM), 2.0 μL dNTPs (2.5 mM), 0.25 μL Taq DNA polymerase (New England BioLabs), and 1.0 μL both forward and reverse primers (25 pM/μL); the final volume was adjusted to 25 μL with sterile distilled water. DNA amplification was performed in an Eppendorf thermocycler using an initial denaturation step at 95 °C for 10 min followed by 30 cycles of amplification (denaturation at 95 °C for 1 min, annealing at 55 °C for 1 min, and extension at 72 °C for 1.5 min), and ending with a final extension at 72 °C for 5 min. The PCR product was visualized by agarose (1.5%) gel electrophoresis and ethidium bromide staining to confirm the predicted 181 base pair band for GFP present in the transconjugant colonies.

### 4.8. Fluorescence-Activated Cell Sorting of Donor and Transconjugant Bacteria

Fecal pellets stored in LB containing 30% glycerol were thawed and processed for isolating bacterial cells using the method described by Ronda et al. 2019 with the following modifications. Fecal pellets were mechanically homogenized with a pestle. A total of 750 μL of PBS was added and vortexed for 15 s before centrifuging at 1000 rpm for 30 s. Then, 500–750 μL of supernatant was carefully removed and placed in a fresh microcentrifuge tube. Next, three more iterations of washing the bacterial suspension were performed by adding and replacing 750 μL of PBS with centrifugation at 1000 rpm for 30 s. The saved supernatant from these four iterations was centrifuged at 6600 rpm for 5 min and the pellet was retrieved. To remove more debris, we did two additional iterations at 1000 rpm for 30 s. The final supernatant was centrifuged at 6600 rpm for 5 min. The pellet was retrieved and resuspended in 500 μL of PBS. All samples were filtered through a 40 μM cell strainer before diluting into filter-sterilized PBS for flow cytometry.

FACS of bacteria was performed at the Flow Cytometry Core Facility at Michigan State University as follows. The BD Influx cell sorter was used to acquire, analyze, and sort donors (mScarlet+/GFP+), transconjugants (GFP+ only), and resident fecal bacteria (mScarlet−/GFP−). The 488 laser (bandpass filter 530/40) and 552 laser (bandpass filter 585/29) were used to detect GFP and mScarlet fluorescing bacteria, respectively. The background was assessed by analyzing fecal samples before gavaging mice with the donor strain and fecal samples collected from the control mice over time. The gating strategy was based on laboratory cultures of positive control bacteria expressing different fluorescent proteins alone and in combination and was effectively used in another study [18]. FCS Express 7 Plus software was used to analyze the FACS data and create plots for the manuscript.

### 4.9. DNA Extraction from FACS Transconjugant Bacterial Cells

Bacterial cells were pelleted by centrifugation at 7500 rpm for 20 min, and the supernatant was discarded. DNeasy Blood & Tissue Kit (QIAGEN; Germantown, MD, USA) was used to extract genomic DNA from FACS cells according to the manufacturer’s instructions. The extracted DNA was immediately stored at −80 °C.

### 4.10. 16S rRNA Gene Sequencing and Analyses

We used a two-step PCR approach based on the primer pair (515-Forward: GTGCCAGCMGCCGCGGTA; and 806-Reverse: GGACTACHVGGGTWTCTAAT) [54] for amplifying the 16S V4 region of bacterial DNA extracted from FACS transconjugant cells. The first round of PCR was performed in our laboratory using dual indexed primers with the underlined tags on the 5′ ends.

CS1-TS-F: 5′—ACACTGACGACATGGTTCTACAGTGCCAGCMGCCGCGGTA—3′

CS2-TS-R:5′—TACGGTAGCAGAGACTTGGTCTGGACTACHVGGGTWTCTAAT—3′

We used approximately 10 nanogram template DNA from each sample in a 25 μL reaction mixture in PCR tubes. Each reaction mixture contained 12.5 μL of DreamTaq PCR Master Mix (ThermoFisher, Catalog # K1071), 1.0 μL of both forward and reverse primers (10 μM), and sterile distilled water to adjust the final volume to 25 μL. DNA amplification was performed in an Eppendorf thermocycler using an initial denaturation step at 95 °C for 2 min followed by 30 cycles of amplification, denaturation at 95 °C for 40 s, annealing at 50 °C for 30 s, and extension at 72 °C for 60 s, ending with a final extension at 72 °C for 7 min. The PCR product was visualized by agarose (1.5%) gel electrophoresis and ethidium bromide staining to confirm amplification of the V4 region. The concentration of PCR product was measured using a Qubit 4 fluorometer (Invitrogen, Waltham, MA, USA), and the samples were normalized to a range from 15 ng/μL to 25 ng/μL.

The primary PCR products prepared and normalized in our laboratory were submitted to the Michigan State University Research Technology Support Facility (MSU RTSF) Genomics Core, where these samples were amplified using primers with the Fluidigm common oligos CS1/CS2 fused to their 5′ ends. The Genomics Core performed secondary PCR using dual-indexed, Illumina-compatible primers, which target the Fluidigm CS1/CS2 oligomers at the ends of our primary PCR products. The PCR reaction recipe and cycling conditions were the same as described above in primary PCR. More details can be found on the MSU RTSF website: https://rtsf.natsci.msu.edu/genomics/sample-requirements/aviti-illumina-short-read-sequencing-sample-requirements.aspx#Other (accessed on 19 December 2024) Amplicons were batch-normalized using a SequalPrep Normalization plate (ThermoFisher Scientific/Applied Biosystems part # A1051001) and the recovered product was pooled. The pool was concentrated using an Amicon Ultra-0.5 Centrifugal Filter Unit (Millipore Sigma part # UFC5050). The pool was screened for quality and quantified using a combination of Qubit dsDNA HS, Agilent 4200 TapeStation HS DNA1000, and Invitrogen Collibri Library Quantification qPCR assays.

This pool was loaded onto one Illumina MiSeq v2 nano flow cell and sequencing was carried out in a 2 × 250 bp paired end format using a MiSeq v2 500 cycle reagent cartridge. Custom sequencing and index primers complementary to the Fluidigm CS1 and CS2 oligomers were added to appropriate wells of the reagent cartridge. Base calling was performed by Illumina Real Time Analysis (RTA) v1.18.54, and the output of RTA was demultiplexed and converted to FastQ format with Illumina Bcl2fastq v2.20.0. A FastQC report of the run output was created to determine the quality scores of the sequenced data.

We analyzed the 16S rRNA gene sequencing data using the QIIME2 16S pipeline [55] on the web-based platform Nephele (National Institutes of Health (NIH), USA) [56]. The Silva 16S ribosomal gene database (version 4) was used for taxonomic assignment of 16S rRNA gene amplicons [57]. Sequences with 97% identity were combined into single operational taxonomic units (OTUs). The relative abundance of each taxon in each sample was calculated and the taxa not comprising at least 0.5% of reads in at least one sample were pruned from the dataset. Diversity indices, principal component analysis (PCA) using the variance–covariance matrix, and SIMPER analyses were calculated using PAST 4.03 [58].

## 5. Conclusions

Utilizing a FMT mouse model harboring human-derived gut microbiota, we demonstrated that a human commensal *E. coli* strain, LM715-1, effectively colonized the mice and mediated the transfer of antibiotic resistance determinants to a diverse array of gut bacterial families without the presence of antibiotic selection pressure. Several bacterial families, including Lachnospiraceae, Clostridiaceae, Pseudomonadaceae, Rhodanobacteraceae, Erysipelotrichaceae, Oscillospiraceae, and Butyricicoccaceae, were identified as putative recipients of the ARG-bearing plasmid.

This FMT mouse model stably colonized with human-derived gut microbiota that transfers to offspring vertically will be very useful for studying the characteristics of ARG transfer into a human microbiome under normal physiological conditions of the gastrointestinal environment. This model enables a detailed investigation of the dynamics of ARG spread and provides comparative insights into conjugation-based HGT in the human gut microbial community. Other human-derived communities could be transplanted into mice of various genetic backgrounds and stably maintained to test the effects of the varied microbiomes and varied genetic backgrounds among other factors such as diet that may affect HGT of ARGs. It thus offers a realistic platform for developing targeted interventions to address the significant threat posed by antibiotic-resistant bacteria through the horizontal gene transfer of ARGs within complex microbial communities.

## Figures and Tables

**Table 1:** These primers were used for the detection of fluorescent markers in the donor and recipient strains.

Primer	Product Size (in bp)	Primer Sequence (5′-3′)	Target Gene	Gene Bank Accession No. References
*gfp*F	182	*ggtgaaggtgaaggtgatgc*	*gfp*	U73901.1
*gfp*R		*cttctggcatggcagacttg*		
*mScarlet*F	371	*cgcgtgatgaactttgaaga*	*mScarlet-I*	KY021424.1
*mScarlet*R		*tcgctgcgttcatactgttc*		

## Data Availability

Supporting data for this manuscript are available on request from the corresponding author.
